# Endoscopic ultrasound-guided transvariceal coil anchoring combined with sandwich injection for giant isolated gastric varices type 1

**DOI:** 10.1055/a-2946-1986

**Published:** 2026-09-24

**Authors:** Zhenkai Gao, Xudong Wen, Chengyang Wu, Xuesong Bai, Xiao Wang

**Affiliations:** 1 Department of GastroenterologySichuan Provincial People's Hospital East Sichuan Hospital & Dazhou First People's HospitalDazhouChina; 2 Department of Gastroenterology, Chengdu Integrated TCM & Western Medicine Hospital 118385 Chengdu University of Traditional Chinese MedicineChengduChina

Endoscopic ultrasound (EUS)-guided coil embolization combined with cyanoacrylate injection is an efficacious modality for the management of gastric varices. 1​ ​2​ However, in cases of giant isolated gastric varices type 1 (IGV1), ectopic embolization due to coil migration remains a concern, particularly when the variceal diameter exceeds the maximum available coil dimensions. 3​ ​4​ Here, we demonstrate a transvariceal cross-wall anchoring technique designed to prevent coil migration ( Video 1 ).

**Video 1** EUS-guided transvariceal coil anchoring combined with lauromacrogol–cyanoacrylate–lauromacrogol sandwich injection achieved effective obliteration and shrinkage of giant isolated gastric varices type 1 without ectopic distribution. Video URI:


A 79-year-old woman presented with hematemesis. Computed tomography and gastroscopy identified giant IGV1 with stigmata of recent bleeding ( Fig. 1 ). EUS revealed multiple cardiofundal varices, with a maximal diameter of 2.2 cm ( Fig. 2a ). Under EUS guidance, a 19-gauge needle was advanced toward the presumed inflow tract. To achieve mechanical anchoring, the needle intentionally traversed the proximal varix and crossed the intervening variceal wall into the adjacent distal varix ( Fig. 2b ). A 10-mm×14-cm coil was slowly deployed across the intervening variceal wall while the needle was gradually withdrawn, releasing half positioned in the distal varix and half in the proximal varix, thereby establishing secure cross-wall anchorage ( Fig. 2c ). A second coil was then deployed entirely within the proximal varix to provide an additional scaffold. Embolization was then performed using a sandwich technique comprising 3 mL of lauromacrogol, 1 mL of 2-octyl cyanoacrylate, and a 3-mL lauromacrogol flush ( Fig. 2d ). Doppler EUS confirmed the immediate cessation of blood flow, and endoscopy showed the collapse of the varices ( Fig. 3 ). No short- or long-term complications occurred. Four-month follow-up gastroscopy showed glue extrusion with ulcer formation at the treated IGV1 site ( Fig. 4 ).

**Fig. 1:**
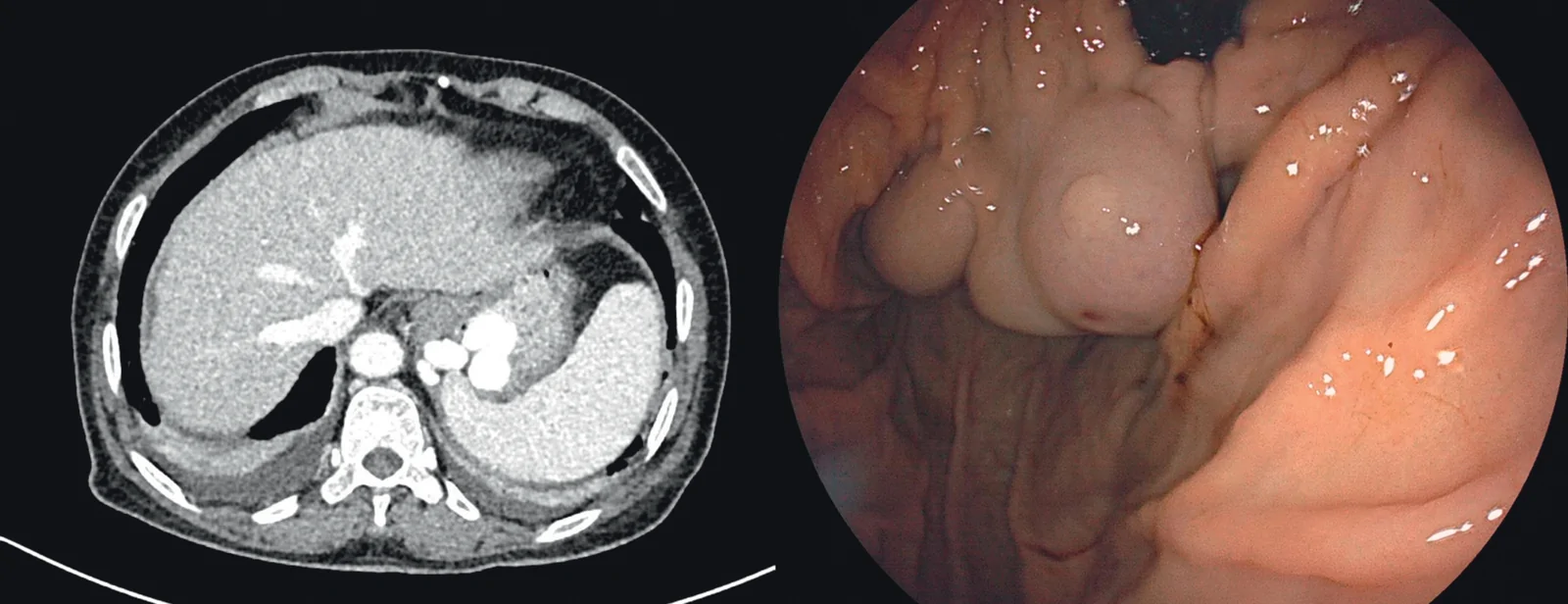
Pretreatment computed tomographic and endoscopic images of isolated gastric varices type 1.

**Fig. 2:**
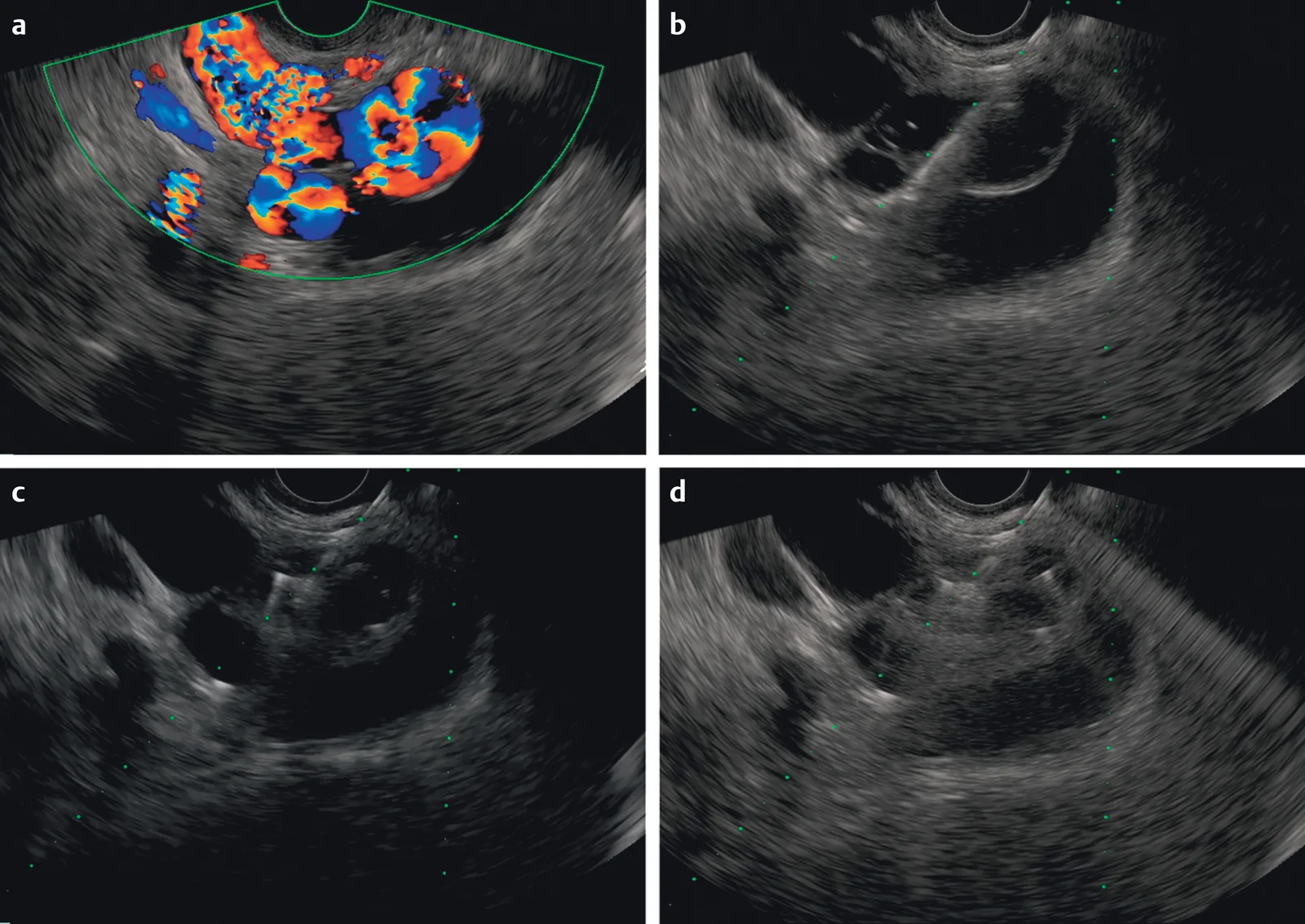
EUS-guided obliteration of giant isolated gastric varices type 1 using a coil-anchoring and sandwich injection technique. ( **a** ) EUS with Doppler imaging revealed multiple intramural fundal varices (> 1.4 cm in diameter) with mixed and abundant blood flow. ( **b** ) Under EUS guidance, the needle traversed the proximal varix and entered the distal varix. ( **c** ) The first coil was deployed across the proximal and distal varices, with one half released into each varix, thereby providing anchoring across the variceal wall. ( **d** ) A second coil was released near the first in the proximal varix, followed by a sandwich injection around the coils.

**Fig. 3:**
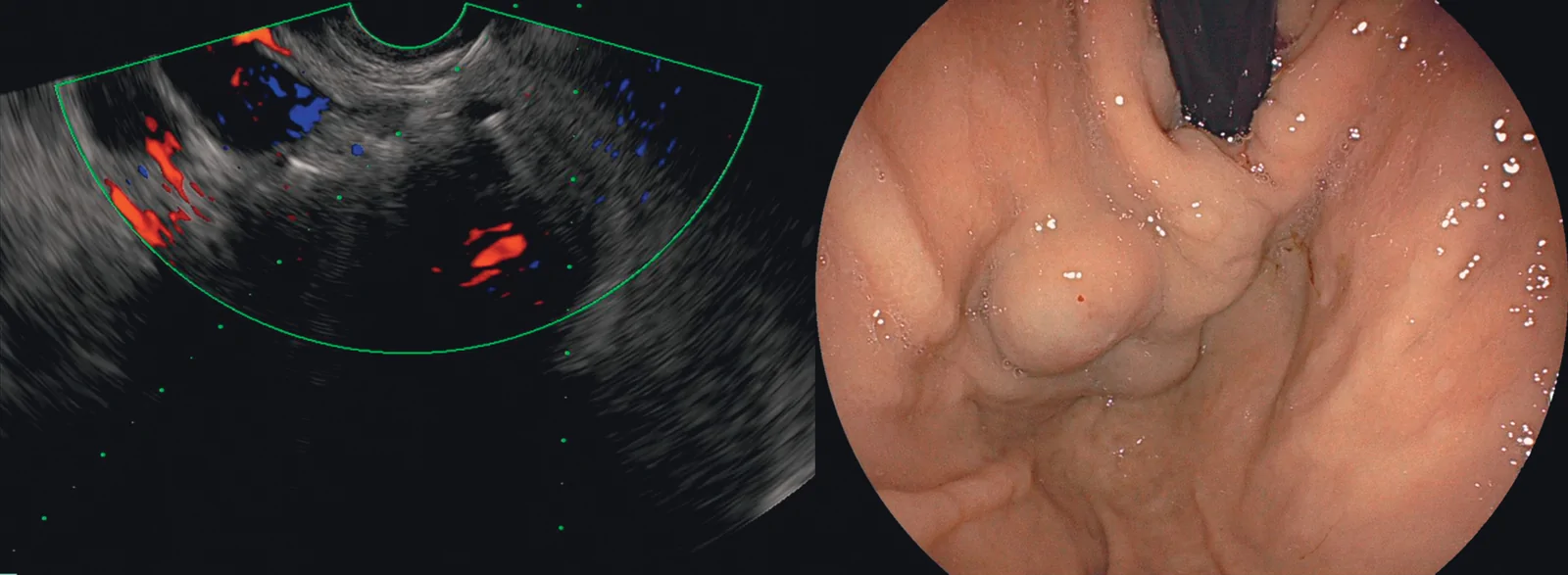
Immediate Doppler imaging revealed no blood flow within the varices and post-treatment endoscopy showed the collapse of the varices.

**Fig. 4:**
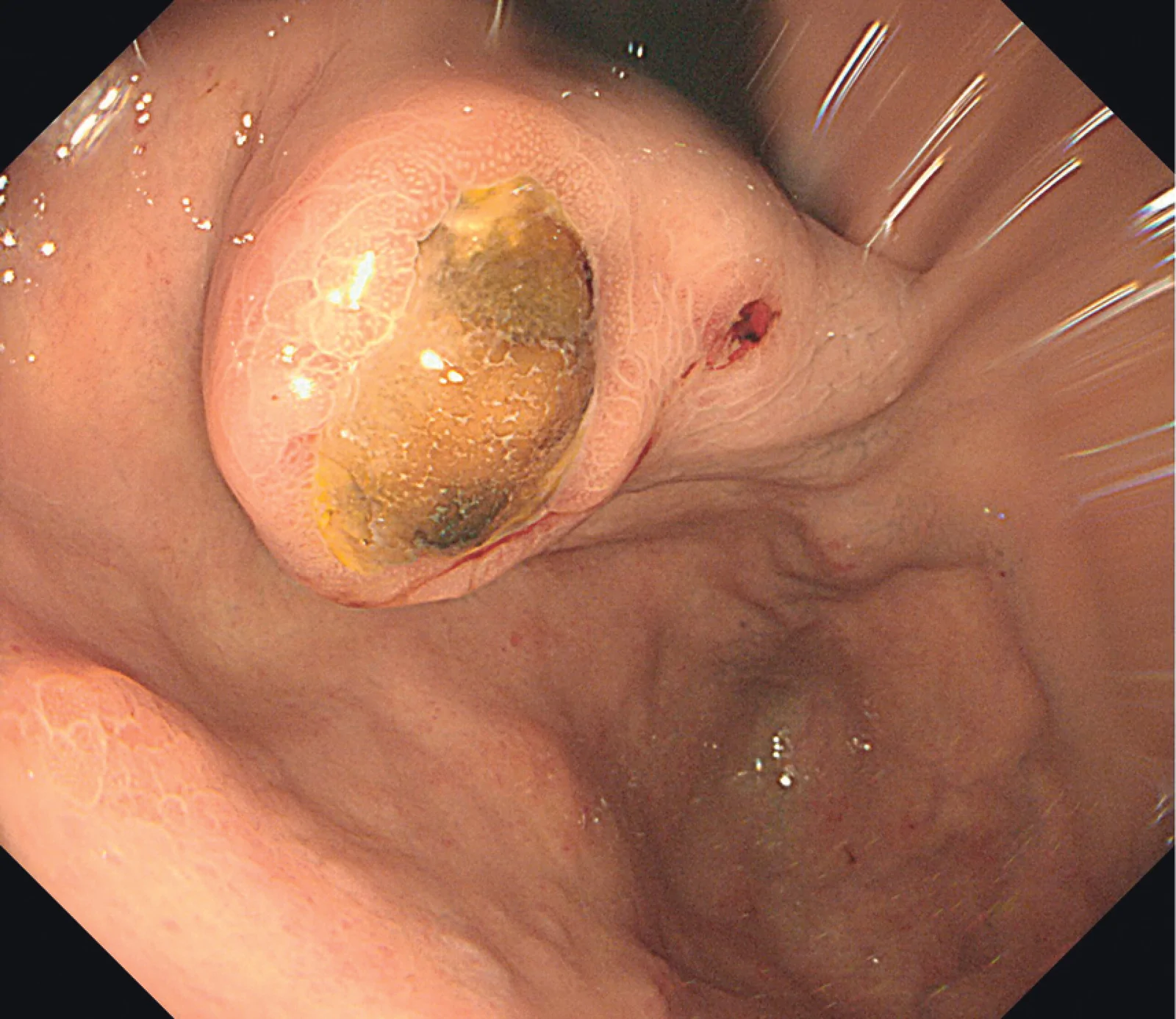
At 4-month follow-up, gastroscopy showed glue extrusion with ulcer formation at the site of the treated IGV1, without evidence of recurrent bleeding.

The transvariceal cross-wall anchoring technique provides a practical solution for managing giant IGV1 ( Fig. 5 ). To achieve the precise anchorage, synchronous slow withdrawal of the puncture needle during deployment is essential. This simple and feasible maneuver may help prevent coil migration and provide a reliable strategy for the simultaneous obliteration of giant IGV1.

**Fig. 5:**
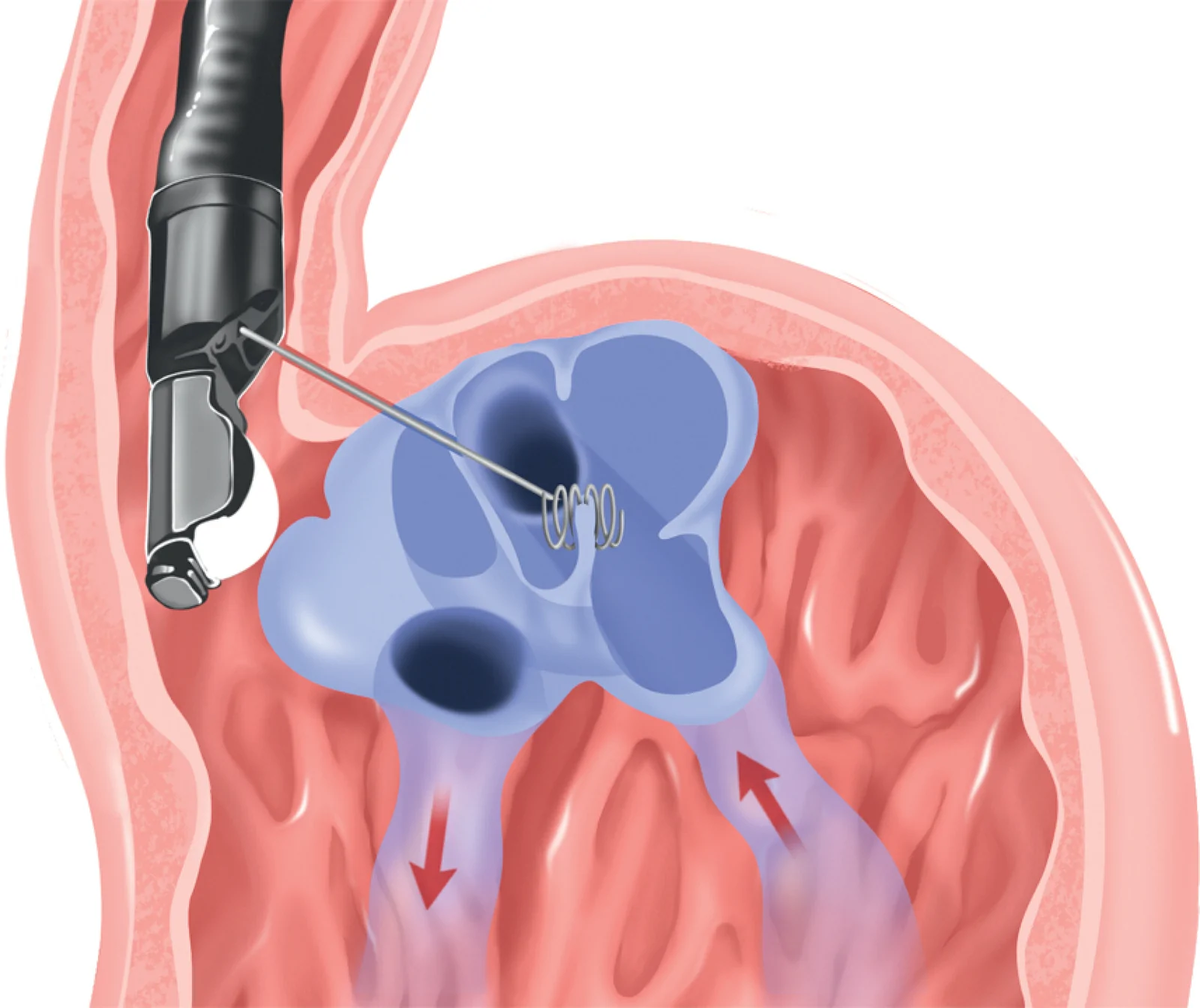
Schematic illustration of EUS-guided transvariceal coil anchoring combined with sandwich injection for giant isolated gastric varices type 1.

Endoscopy_UCTN_Code_TTT_1AS_2AL
